# Cytotoxicity and Antiangiogenic Activity of *Turbinaria ornata* Agardh and *Padina australis* Hauck Ethanolic Extracts

**DOI:** 10.1155/2018/3709491

**Published:** 2018-08-06

**Authors:** Jenefa L. Canoy, Jayzon G. Bitacura

**Affiliations:** Department of Biological Sciences, Visayas State University, Visca, 6521-A Baybay City, Leyte, Philippines

## Abstract

Brown macroalgae species are constantly reported as potential sources of bioactive compounds useful in inhibiting cell proliferation and vascular formation. Thus, this study was conducted to determine and compare the *in vitro* cytotoxic activities of *Turbinaria ornata* Agardh and *Padina australis* Hauck ethanolic extracts against baker's yeast (*Saccharomyces cerevisiae*) using the resazurin reduction test (RRT) and investigate their *in vivo* antiangiogenic activity through duck (*Anas platyrhynchos*) chorioallantoic membrane (CAM) assay. Both *T. ornata* and *P. australis* ethanolic extracts exhibited cytotoxic activities at IC_50_ of 530.53 ppm and 528.78 ppm, respectively, and significant cytotoxicity was determined in 750 ppm and 1000 ppm concentrations of *T. ornata* and 1000 ppm concentration of *P. australis*. Also, both *T. ornata* and *P. australis* ethanolic extracts exhibited antiangiogenic activity (100% vascular inhibition) as all the concentrations of both species caused severe vascular damage in all the duck CAM samples treated. These results show the potential future application of these species for cytotoxic activities and vascular inhibition. The conduct of further tests using other model systems is recommended.

## 1. Introduction

Cellular proliferation is an important aspect of disease progression like cancer [1]. Angiogenesis, on the other hand, is the process of new blood vessel growth, which is involved in many pathological and physiological situations. Antiangiogenic therapy has become established as a strategy for the prevention of some illnesses, and many studies have been conducted on angiogenesis inhibitors because the aggravation of some pathogenesis, such as cancer, atherosclerosis, and diabetic retinopathy, is known to depend on the angiogenic phenotype [2]. Many food compounds have been believed to have advantage for human health due to their anticarcinogenic activity. Therefore, the cytotoxic and antiangiogenic activity of food components like brown macroalgae has received increased attention nowadays [3].

Macroalgae, also known as seaweed, is one of the most extensively used functional foods and medicinal herbs in many parts of the globe especially in Asian countries. It is known as functional food because of its richness in lipids [4], minerals [5], and certain nutrients [6]. It also has several bioactive substances like polysaccharides [7], proteins [8], and polyphenols [9], with potential medicinal uses against cancer [10], inflammation [11], allergy [12], diabetes [13], thrombosis [14], obesity [15], lipidemia [16], hypertension [17], and other degenerative diseases.

Brown macroalgae species are widely studied for their potential pharmaceutical use. They are found to have antioxidant [18], antidiabetic [19, 20], anti-inflammatory [21], antiviral [22], antiproliferative [23], and anticoagulant [24] properties. The brown seaweeds are studied because they are rich in fucoidan and fucoxanthin [25]. These compounds are constantly reported to possess cytotoxic and antiangiogenic activities [26, 27]. *Sargassum* species are frequently studied for their antiangiogenic activity [23, 28, 29]. However, they are presently being regulated for harvest [30]. Thus, there is a need to screen more species of brown macroalgae for their cytotoxic and antiangiogenic effects.

Two of the common brown macroalgae species found in the coastal areas of Baybay City, Leyte, are *Turbinaria ornata* Agardh and *Padina australis* Hauck. As of the present knowledge, these species have only been studied for their anti-inflammatory [31, 32] and antibacterial [33, 34] activities. Thus, this study explored the potential of these two brown macroalgae species for their cytotoxicity and antiangiogenic activities.

## 2. Materials and Methods

### 2.1. Seaweed Collection

Around 100–200 g of *T. ornata* and *P. australis* was collected from the coastal areas of Maitum and Punta, Baybay City, Leyte, respectively, in March 2017. It is at this time when these seaweed species are at their mature stages and are in abundance in the areas. Upon collection, the seaweeds were washed thoroughly with seawater to remove epiphytes and other debris [19] and were then brought to the laboratory of the Department of Biological Sciences, Visayas State University.

### 2.2. Seaweed Identification

Identification of the seaweed species was based on the morphological analyses of the thalli (blades, holdfasts, etc.) following the book of Trono [35]. Prof. Dr. Humberto R. Montes, Jr., and Prof. Julissah C. Evangelio of the Institute of Tropical Ecology and Environmental Management and Department of Biological Sciences, Visayas State University, then validated the identifications. Voucher specimens were also deposited at the VSU Herbarium.

### 2.3. Ethanolic Extraction and Preparation of Concentrations

In the laboratory, the cleaned seaweeds were rinsed with tap water to remove excessive salts and were then oven-dried at 50°C for 36–72 h [19]. The dried samples were powdered using mortar and pestle. Ten grams (10 g) of powdered samples was soaked in 100 mL of 100% ethanol for 24 h and was filtered using a filter paper (Whatman number 1, Sigma-Aldrich Inc.). The filtrate (crude ethanolic fraction) was concentrated at 40–50°C using a rotary evaporator (RV10, IKA®) [36]. A 1000 ppm stock solution was prepared by dissolving 0.1 g of the extract in 100 mL of sterile distilled water. Concentrations of 750 ppm, 500 ppm, 250 ppm, and 100 ppm were then made from this solution.

### 2.4. Cytotoxicity Assay

The cytotoxicity of *T. ornata* and *P. australis* extracts was assayed through the resazurin reduction test (RRT) following the method of Tualla and Bitacura [37] with slight modification. This test is based on the ability of living cells to reduce the blue resazurin into pink resorufin. Treatments included in the assay were the different concentrations of *T. ornata* and *P. australis* ethanolic extracts. For the control set-up, distilled water served as the negative control while 0.1 M of CdCl_2_ as the positive control. One point five grams (1.5 g) of baker's yeast was activated by dissolving it in 40 mL sterile distilled water. This mixture was then diluted at 1 : 100. Fifty microliters (50 *μ*L) of baker's yeast cell suspension was pipetted and placed into the 96-well sterile microplate in triplicate. Then, 50 *μ*L of the treatments was added, and the cells were then incubated for an hour. Then, 10 *μ*L of resazurin solution was added into the wells starting from the negative control up to the positive control. After 12 h, changes in color were observed and the absorbance of the different treatments was determined at 630 nm using a microplate reader (Heales 580 MB, Shenzhen Heales Technology Development Co. Ltd.) at the National Coconut Research Center (NCRC) Laboratory, Visayas State University, and the % cytotoxicity of the treatments was computed using the following equation [38]:

(1) $$\begin{array}{c} \%cytotoxicity=\frac{abs.\text{ of the }treatment\;at\;630\;nm}{abs.\text{ of the }positive\;control\;at\;630\;nm}\times 100. \end{array}$$

### 2.5. Antiangiogenic Assay

The chorioallantoic membrane (CAM) assay was used in order to determine the antiangiogenic activity of the treatments. It was performed according to the method of Gururaj et al. [39] with modifications. Fertilized duck eggs were obtained from a commercial supplier in Brgy. Maganhan, Baybay, Leyte. Day 0 eggs (3 replicates) were placed in an incubator at 37°C at the Department of Animal Science, Visayas State University, Baybay, Leyte.

At the fifth day of incubation, the eggs were candled and inspected to determine the egg viability and the position of the embryo. The center of the eggshell outside the air sac was disinfected with 0.1% benzalkonium bromide by wiping its surface using sterile cotton. A 10 mm diameter window was gently drilled at the blunt end (air space) of the egg using a sterile dissecting needle. Two drops of 10% NaCl solution were added adjacent to the CAM to moisten the inner shell membrane to easily separate the membrane from the CAM. The membrane and the CAM were separated without force after being clamped and raised using forceps. A window of 1 cm diameter was sectioned on the membrane to expose the vascular zone. Sterilized filter paper disks with a diameter of 7 mm were individually loaded with 5 *μ*L of seaweed ethanolic extracts and sterile distilled water (negative control). These were directly adhered to the vascular zone with the right density of blood vessels [40, 41]. The inoculated CAMs were resealed with 3^″^ × 3^″^ sterile plastic and returned to the incubator and was allowed to further develop [28]. After 24 h, the eggs were reopened.

The CAM area was visually assessed for vascular damage. Representative areas or fractal segments were observed and photodocumented. The CAMs were scored using the CAM scoring guide by Raga et al. [42] with slight modification with 6 being the highest and 0 being the lowest score. CAM scores were set as follows: (6) severe—blood vessels are completely damaged and are not visible anymore, (5) moderate—more than half of the vessel has been damaged, (4) slightly damaged—less than half of the vessel has been damaged, (3) minimal—small fractions are damaged, (2) hemorrhaging—increased blood flow is seen in capillaries, (1) ghost vessels—the capillary is already devoid of blood flow, and (0) no effect at all. Any damage on vasculature and obstruction to normal blood flow were considered positive antiangiogenic effect. Vascular inhibition (VI) was computed using the following formula:

(2) $$\begin{array}{c} \%VI=\frac{number\text{ of }blood\;vessels\;after\;treatment}{number\text{ of }blood\;vessels\;before\;treatment}\times 100. \end{array}$$

### 2.6. Experimental Design and Analysis

This study followed a completely randomized design (CRD). One-way analysis of variance (ANOVA) was used to determine the significant difference of the treatments. Post hoc comparison was used to cluster the various treatments following Tukey's HSD. The results were considered significant at *p* ≤ 0.01. Means were reported as mean ± SE.

## 3. Results and Discussion

**Figure 1:**
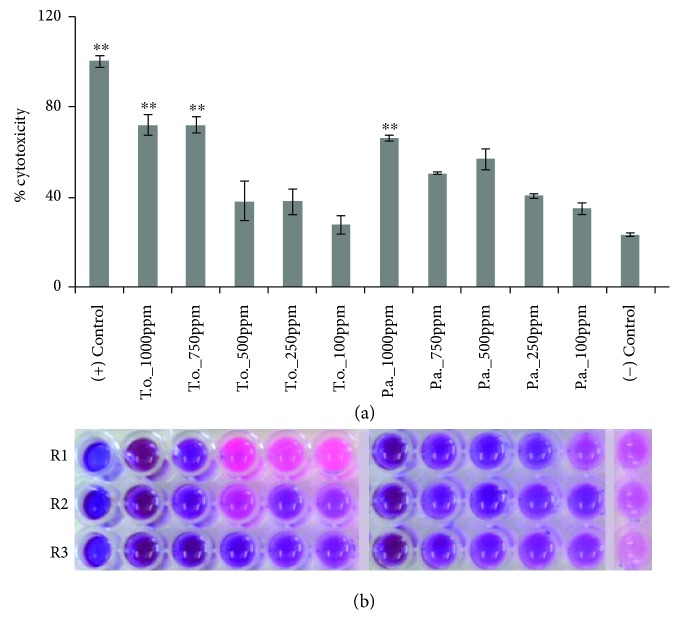
Cytotoxic activity of *T. ornata* and *P. Australis* ethanolic extracts on baker's yeast. (a) Comparison of the percent cytotoxicity (mean ± SE) of different concentrations of brown macroalgae ethanolic extracts against the positive control (0.1 M CdCl_2_) and the negative control (sterile distilled water). ^∗∗^*p* ≤ 0.01 indicates high significance (HSD). (b) Variations in color reactions in the triplicate wells of the different treatments after RRT. T.o.: *Turbinaria ornata*; P.a.: *Padina australis*.

Results of the RRT conducted showed that *T. ornata* and *P. australis* ethanolic extracts possess cytotoxic activity against yeast cells (Figures 1(a) and 1(b)). As shown in Figure 1(b), the cells treated with majority of the concentrations of the seaweed extracts did not change in color just like those treated with 0.1 M CdCl_2_ (positive control). This cytotoxic effect is only attributed to the seaweed extracts since the solvent used in preparing the different concentrations (negative control) did not exhibit cytotoxicity to the treated cells.

Since the variation in the colors of the different treatments was evident, it was decided to quantify the percent cytotoxicity of the treatments through their absorbance at 630 nm. This quantified the amount of resazurin present in the treatment wells. This means that when the yeast cells are viable, they will be able to reduce blue resazurin to pink resorufin giving low absorbance at 630 nm. On the other hand, when the treatments are toxic, there is a lesser ability of the yeast cells to reduce resazurin to resorufin giving higher absorbance at 630 nm. Analysis revealed that strong cytotoxic activities were exhibited by 1000 ppm and 750 ppm of *T. ornata* and 1000 ppm of *P. Australis* ethanolic extracts (Figure 1(a)). These are the treatments that showed highly significant difference against the negative control and no significant difference against the positive control.

**Figure 2:**
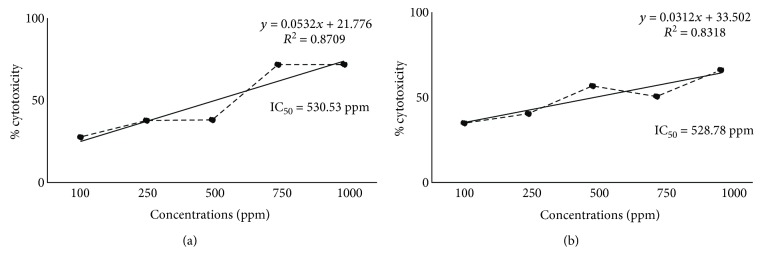
IC_50_ computations for the comparison of the % cytotoxicity between *T. ornata* (a) and *P. australis* (b) under different concentrations of ethanolic extracts.

Furthermore, in order to compare the cytotoxicity of the two brown macroalgae species, their concentrations that could kill 50% of the treated cells (IC_50_) were determined (Figure 2). IC_50_ values for *T. ornata* and *P. australis* on yeast cells were determined at 530.53 ppm and 528.78 ppm, respectively. Lower IC_50_ value means high cytotoxicity of the seaweed ethanolic extracts against yeast cells. The difference in the IC_50_ values of the ethanolic extracts of *P. australis* and *T. ornata* is very slim which could mean no difference in their cytotoxic activity at all.

Moreover, the result of the CAM assay revealed 100% vascular inhibition on all concentrations of both seaweed ethanolic extracts. These results were significantly different with those treated with only sterile distilled water (negative control). This implies that the antiangiogenic activity of the different seaweed concentrations is solely attributed to the extracts and not to the solvent used (Table 1). However, it is believed that a trend could be observed in the antiangiogenic activity if the CAMs are subjected to much lower concentrations of the extracts.

**Figure 3:**
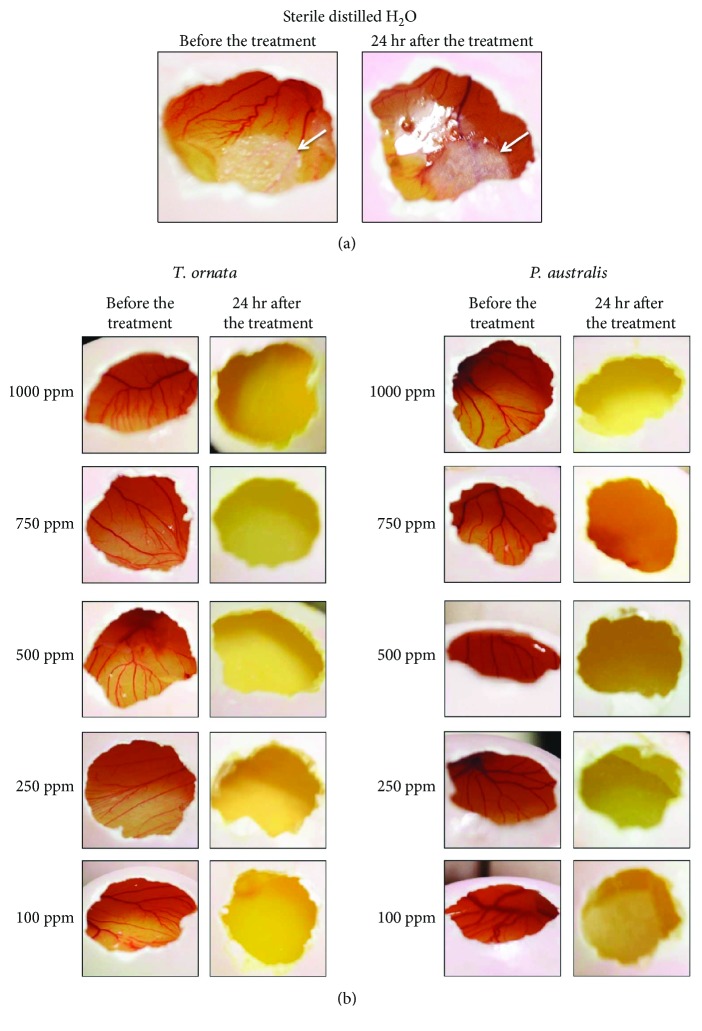
Representative samples of duck CAM before and after treatment of (a) sterile distilled water as the negative control (the arrow points to the placement of the filter paper disks impregnated with the treatment) and (b) different concentrations of *T. ornata* and *P. australis* ethanolic extracts.

It was observed that treatment of CAM blood vessels with sterile distilled water (negative control) shows no effect or any damage on the blood vessels (Figure 3(a)), hence attaining a score of 0 (Table 1). On the other hand, the blood vessels were severely damaged and were not visible anymore after 24 hours of administration of *T. ornata* and *P. australis* ethanolic extracts in all concentrations (Figure 3(b)), hence attaining a score of 6 (Table 1). This means that the ethanolic extracts of both seaweeds possess antiangiogenic activity but were not elaborately compared because the effects were uniform in all the concentrations. In the CAM, angiogenic and angiostatic responses to promoters or inhibitors, respectively, are readily visible. When angiogenic substances are administered to the CAM, there is a visible increase in the density of blood vessels around the implant. On the other hand, administering an antiangiogenic substance into the CAM will make vessels become less dense around the implant, and eventually these vessels disappear [43].

The cytotoxic and antiangiogenic activities of *T. ornata* and *P. australis* could be due to the presence of phenolic compounds, fucoxanthin and fucoidan, in these brown macroalgae species as previously reported [44, 45]. Phenolic compounds have antioxidant properties which are important in combatting the effects of oxidative stress-influenced cancer development involving malignant transformation due to DNA mutations. Namvar et al. [23] reported that cytotoxic activity of the brown algae is positively correlated with its total phenolic contents.

On the other hand, fucoxanthin is a well-known example of natural pigment for anticancer activity [46]. Other research reported on free radical-scavenging activity of fucoxanthin as suggested to be the underlying mechanism for its cytotoxic effect [46]. And an *in vitro* and *in vivo* study done by Kim and colleagues [47] showed that fucoxanthin displays inhibitory effect on tumor growth on B16F10 cells. Another study showed that fucoxanthin has cytotoxic effect and strong antitumor potential as exhibited by its inhibitory effect on liver tumorigenesis [48]. Sugawara et al. [29] also reported that fucoxanthin derived from brown seaweed has the antiangiogenic properties via restraining tube formation of the endothelial cells of the umbilical vein.

Furthermore, fucoidan has been constantly reported for its ability to inhibit tumor formation and metastasis [49, 50]. A study of Koyanagi et al. [51] showed that fucoidan possessed strong inhibitory effect on tube formation of the human umbilical vein, while another study showed significant antiangiogenic activity on human uterine carcinoma HeLa cells suggesting that the antiangiogenic effects may be explained, at least partially, by the antioxidative potential of fucoidan extracts [52]. However, a study conducted by Liu et al. [53] revealed that fucoidan from *Undaria pinnatifida*, also a brown macroalgae species, has restraining effects on cellular proliferation and immigration and the formation of the vessel network and that it also decreased the growth of the blood vessels and decreased the expression of VEGF-A angiogenesis factor.

Fucoxanthin, similar to fucoidan, is not uniform, and its structure highly differs on the species source of isolation; hence, different species of brown macroalgae exhibit different cytotoxic and antiangiogenic activities [21, 46, 54]. One study, for instance, showed that *T. ornata* possesses free radical-scavenging properties due to the high polyphenol content (fucoidan and fucoxanthin) [55], while a study on *P. australis* also revealed the seaweeds' antioxidant activity which is attributed to the presence of different secondary metabolites such as phenolic compounds and carotenoids further suggesting that the mechanism could be due to their individual or collective participation [56].

Other cytotoxicity studies on macroalgae extracted using various polar solvents reveal different cytotoxic activities of seaweed extracts against cell lines. A study conducted by Tantengco et al. [57] reveals that crude extracts of *Kappaphycus alvarezii* and *Eucheuma denticulatum* have strong inhibitory concentrations at 42.62 ppm and 44.12 ppm against human cancer cell lines, respectively. Another study by Spavieri et al. [58] showed active cytotoxicity of *Halidrys siliquosa* and *Bifurcaria bifurcata* on *Trypanosoma brucei* with IC_50_ values of 1.2 and 1.9 *μ*g/mL, respectively. The same difference in cytotoxic activities was observed from extracts of brown macroalgae species. This denotes further that inhibitory concentrations of seaweed extracts against different cell lines or model organisms also differ.

## 4. Conclusion

The ocean has been considered a rich source of compounds possessing novel structures and biological activities. Biologically active molecules isolated from marine organisms have been explored for their applications in pharmaceuticals, nutritional supplements, cosmetics, agrochemicals, molecular probes, and enzymes. This study likewise shows that *T. ornata* and *P. australis* ethanolic extracts have cytotoxic and antiangiogenic properties. These results imply that *T. ornata* and *P. australis* ethanolic extracts have the potential future application for inhibiting cell proliferation and vascular formation. It is recommended however that lower concentrations of the extracts be tested to acquire the IC_50_ for their antiangiogenic activities. The use of other solvents, tests (i.e., MTT assay), and other model systems (i.e., rat aorta) in further studies is recommended as well.

## Figures and Tables

**Table 1:** Antiangiogenic activity of *T. ornata* and *P. australis* ethanolic extracts as revealed by the CAM assay.

Treatments		Vascular inhibition (%)	Vascular damage score	Description of vascular damage
Negative control (sterile distilled water)		0	0	No vascular inhibition
*T. ornata*	1000 ppm	100	6	Severely damaged
	750 ppm	100	6	Severely damaged
	500 ppm	100	6	Severely damaged
	250 ppm	100	6	Severely damaged
	100 ppm	100	6	Severely damaged
*P. australis*	1000 ppm	100	6	Severely damaged
	750 ppm	100	6	Severely damaged
	500 ppm	100	6	Severely damaged
	250 ppm	100	6	Severely damaged
	100 ppm	100	6	Severely damaged

## Data Availability

The data used to support the findings of this study are available from the corresponding author upon request.
